# Delphi-Consensus Weights for Ischemic and Bleeding Events to Be Included in a Composite Outcome for RCTs in Thrombosis Prevention

**DOI:** 10.1371/journal.pone.0018461

**Published:** 2011-04-07

**Authors:** Agnes Dechartres, Pierre Albaladejo, Jean Mantz, Charles Marc Samama, Jean-Philippe Collet, Philippe Gabriel Steg, Philippe Ravaud, Florence Tubach

**Affiliations:** 1 UMR-S 738, INSERM, Paris, France; 2 UMR-S 738, Université Paris Diderot, Paris, France; 3 Université Paris Descartes, Paris, France; 4 Centre d'Epidémiologie Clinique, Hôpital Hôtel-Dieu, APHP, Paris, France; 5 Pôle d'Anesthésie et de Réanimation, Centre Hospitalier Universitaire, Grenoble, France; 6 Université Joseph Fourier, Grenoble, France; 7 Service d'anesthésie réanimation et SMUR, Hôpital Beaujon, APHP, Clichy, France; 8 Université Paris Diderot, Paris, France; 9 Service d'anesthésie réanimation, Hôpital Hôtel-Dieu, APHP, Paris, France; 10 Service de cardiologie, Hôpital Pitié-Salpétrière, APHP, Paris, France; 11 Université Pierre et Marie Curie, France; 12 INSERM U-698, Paris, France; 13 Service de cardiologie, Hôpital Bichat-Claude Bernard, APHP, Paris, France; 14 CIE 801, INSERM, Paris, France; 15 Département d'Epidémiologie, Biostatistique et Recherche Clinique, Hôpital Bichat-Claude Bernard, APHP, Paris, France; Georgetown University Medical Center, United States of America

## Abstract

**Background and Objectives:**

To weight ischemic and bleeding events according to their severity to be used in a composite outcome in RCTs in the field of thrombosis prevention.

**Method:**

Using a Delphi consensus method, a panel of anaesthesiology and cardiology experts rated the severity of thrombotic and bleeding clinical events. The ratings were expressed on a 10-point scale. The median and quartiles of the ratings of each item were returned to the experts. Then, the panel members evaluated the events a second time with knowledge of the group responses from the first round. Cronbach's a was used as a measure of homogeneity for the ratings. The final rating for each event corresponded to the median rating obtained at the last Delphi round.

**Results:**

Of 70 experts invited, 32 (46%) accepted to participate. Consensus was reached at the second round as indicated by Cronbach's a value (0.99 (95% CI 0.98-1.00)) so the Delphi was stopped. Severity ranged from under-popliteal venous thrombosis (median = 3, Q1 = 2; Q3 = 3) to ischemic stroke or intracerebral hemorrhage with severe disability at 7 days and massive pulmonary embolism (median = 9, Q1 = 9; Q3 = 9). Ratings did not differ according to the medical specialty of experts.

**Conclusions:**

These ratings could be used to weight ischemic and bleeding events of various severity comprising a composite outcome in the field of thrombosis prevention.

## Introduction

A composite outcome consists of two or more component outcomes. Patients who have experienced any one of the events specified by the components are considered to have experienced the composite outcome[1], [2]. The use of composite outcomes in RCTs is common, particularly in cardiology[3] having the advantage of reducing sample size requirement, costs and time because of higher event rates. Composite outcomes estimate the net clinical benefit of treatment and enable to avoid an arbitrary choice between a number of important outcomes[2], [4]–[7] so they may be used to summarize the risk/benefit profile of an intervention[8], [9]. In the field of thrombosis prevention where treatments aim to decrease the rate of ischemic events but may cause hemorrhagic side effects of various severity, using composite outcomes including both ischemic and hemorrhagic events may be particularly appropriate to capture the net clinical benefit. Many authors have argued that all components of a composite outcome should be of similar importance to adequately interpret treatment effect[1]–[4], [6]–[8], [10]–[12] which is not frequently the case. Cordoba showed that the components were not of similar importance in 70% of RCTs reporting a binary composite outcome[1]. Choosing individual components of the same importance might also be irrelevant if the aim is to capture the overall impact of treatment. This is why some authors have proposed to assign each component a weight reflecting severity[8], [12]–[14]. Since weighting may be somewhat arbitrary, it should be subjected to consensus panel[12], [13], [15].

STRATAGEM is a multicenter, randomized, double-blind, placebo-controlled trial whose objective was to compare low-dose aspirin therapy versus placebo (stopping anti platelet therapy) in the perioperative period in patients treated with antiplatelet therapy as secondary prevention (with documented symptomatic stable atherothrombotic disease) who undergo non-coronary surgery (registration number: NCT00190307, IRB authorization from the “Comité Consultatif de Protection des Personnes se prêtant à la Recherche Biomédicale (CCPPRB) de Paris Bichat” (Ref 2004/18, authorization obtained the 10th of Novembre 2004). The composite outcome took into account the balance of risk and benefit associated with maintaining antiplatelet therapy in the peri-operative period including both ischemic events (e.g., ischemic stroke, non-fatal myocardial infarction, acute limb ischemia, clinical deep venous thrombosis) and bleeding events (e.g., life-threatening bleeding or conducive to revision, or redo surgery, cerebral hemorrhage, intra- or retroperitoneal bleeding, bleeding requiring the transfusion of more than 3 units of packed red blood cells) in addition to overall mortality within one month following surgery. Since the individual components of this composite outcome clearly do not have the same value and severity, the aim of the present project was to attribute consensus-driven weights to ischemic and bleeding events according to their severity to be used in a composite outcome in RCTs in the field of thrombosis prevention.

## Methods

### Study design

The Delphi method was used to synthesize expert opinion [16], [17]. It is a well-recognized method to reach consensus, relying on the following principles: anonymity, iteration, controlled feedback, and statistical aggregation of group responses [18]–[20].

### Staff

A steering committee was initiated to perform this study and included all authors. The committee was responsible for the selection of events to be evaluated and experts, the analysis of the responses and the presentation of results.

### Selection of experts

Experts were recruited from clinical disciplines involved in the management of patients with atherothrombotic disease in the perioperative period. In France, both cardiologists and anesthesiologists are involved in this field. Experienced academic experts were identified from different centers all over the country within national organizations such as the French Society of Anesthesia and Intensive Care or the French Society of Cardiology. The selected experts had also to be involved in design, execution and evaluation of clinical trials. Thirty cardiologists and 40 anaesthesiologists were invited to participate in the study. The experts were sent a standardized information package containing a synopsis of the study and a description of the Delphi process. The experts were informed that the consensus-driven ratings would be used as weights in a composite outcome.

### Selection of events to be evaluated

Events to be evaluated were identified from the Common Terminology Criteria for Adverse Events (CTCAE) v3.0[21] which is a descriptive terminology that can be used for Adverse Event (AE) reporting. A grading (severity) scale is provided for each AE term. One author (F.T.) identified 28 ischemic and bleeding events that were then submitted to the steering committee for validation to enter the first Delphi round. They covered all the fields addressed by the STRATAGEM composite endpoint, in a more detailed way (for instance myocardial infarction was addressed by 3 different events corresponding to 3 different levels of severity in accordance with the CTCAE). We did not include death among the events to be assessed since the steering committee decided to attribute it automatically the worse rating (i.e., 10). The items involved in the Delphi process are reported in table 1.

**Table 1:** Delphi panel events.

Clinical events	Definition
***Ischemic events***	
Transient ischemic attack	Transient ischemic event q 24 hrs duration
Ischemic stroke with no symptom at 7 days	Defined by a modified Rankin scale of 0–1
Ischemic stroke with slight disability at 7 days	Defined by a modified Rankin scale of 2
Ischemic stroke with moderate disability at 7 days	Defined by a modified Rankin scale of 3
Ischemic stroke with severe disability at 7 days	Defined by a modified Rankin scale of 4–5
Limb ischemia not requiring heparin or intervention	Brief (q 24 hrs) episode of ischemia managed non surgically and without permanent deficit
Limb ischemia requiring heparin or intervention	Recurring or prolonged (> 24 hrs) requiring medical or surgical intervention
Limb ischemia requiring amputation*	Life-threatening, disabling limb ischemia requiring end organ damage (i.e., limb loss)
Increased level of troponin	Without new Q wave or heart failure
Non-fatal myocardial infarction without heart failure	Killip 1
Non-fatal myocardial infarction with heart failure	Killip ≥2
Under-popliteal deep venous thrombosis	Under-popliteal deep venous thrombosis
Deep venous thrombosis with iliac extension	Deep venous thrombosis with iliac extension
Venous thrombosis of the pectoral limb	Venous thrombosis of the pectoral limb
Venous thrombosis other	Venous thrombosis other
Pulmonary embolism	Pulmonary embolism
Massive pulmonary embolism	Clinical or echographical acute pulmonary heart and/or impact on hepatic biology and/or more than half obstruction at angiography
***Hemorrhagic events***	
Intracerebral hemorrhage with no symptom at 7 days	Defined by a modified Rankin scale of 0–1
Intracerebral hemorrhage with slight disability at 7 days	Defined by a modified Rankin scale of 2
Intracerebral hemorrhage with moderate disability at 7 days	Defined by a modified Rankin scale of 3
Intracerebral hemorrhage with severe disability at 7 days	Defined by a modified Rankin scale of 4–5
Bleeding with increased length of stay	Mild bleeding not requiring intervention other than iron supplements
Bleeding requiring redo surgery or endoscopic sclerosis	
Bleeding requiring both redo surgery and interventions to maintain cardiac output	Bleeding with life-threatening consequences requiring urgent and major interventions
Bleeding requiring transfusion of 3 U or more packed red blood cells	Bleeding requiring transfusion of 3 U or more packed red blood cells
Bleeding requiring both transfusion of 3 U or more packed red blood cells and interventions to increase cardiac output	Bleeding with life-threatening consequences requiring urgent and major interventions
Intra or retroperitoneal bleeding	Intra or retroperitoneal bleeding
Intra or retroperitoneal bleeding requiring interventions to maintain cardiac output	Bleeding with life-threatening consequences requiring urgent and major interventions

Mesenteric ischaemia is considered as peripheral ischemia and then classified as “Life-threatening, disabling limb ischemia requiring end organ damage (i.e., limb loss)” and thus with limb ischemia requiring amputation.

### Delphi consensus

The steering committee planned to perform at least two Delphi rounds. If consensus was not reached after 2 rounds, it was planned to perform additional rounds until a consensus was reached. The consensus process was conducted via email. Two reminders were sent at each round in case of non response.

In the first Delphi round, each member of the panel evaluated the severity of each of the 28 events on a 10-point scale. For each event, the experts were asked to answer the following question: “According to you, how severe is this event?”. A 10-point scale with the anchors “not severe at all” at 0 and “extremely severe” at 9 was used to record the responses. The experts had the possibility to suggest events that were missing. They were added at the following round provided that they were not redundant with the other events. The median rating (1^st^ quartile-3^rd^ quartile (Q1–Q3)) for the whole group was established for each individual event.

In the second round, the experts considered the same event, and were also informed of each event rating at the first round by reporting of the median ((Q1–Q3)) rating on the scale for each event. The experts were asked to rate each event again in light of the responses at the first round.

### Analysis

For each event, the experts' ratings were summarized as median (Q1–Q3). We applied a Last Observation Carried Forward (LOCF) strategy for missing data after the first round that is to say that, if an expert did not answer the second round, we considered his answers at the first round.

The concept of consensus within a group was defined as homogeneity or consistency opinion among the experts. Assuming that each event was characterized by a constant but unknown severity, the ratings of the experts could be considered as multiple measures of this characteristic. We used Cronbach's a to measure internal consistency among the experts for the set of events reflects the extent of consensus within the group for the severity of the set of events. When Cronbach's a is close to 1.0, it can be argued that there is consistency in the responses of the index panel, suggesting consensus. According to the recommendation of Bland and Altman [22], we considered that a consensus would be reached for a Cronbach's a value of 0.95. We also calculated intra-class correlation coefficient as a measure of the overall agreement between experts [23]. Ninety five percent confidence intervals for both Cronbach's a and intra-class correlation coefficient were calculated with bootstraps (1000 simulations). We planned to stop the Delphi consensus after the second round if the Cronbach's a value was superior to 0.95. The final weight for each event was the median rating obtained at the last Delphi round.

All analyses were performed on R version 2.10.0[24].

## Results

### Delphi process

Of the 70 experts invited (30 cardiologists and 40 anaesthesiologists), 32 (46%) accepted to participate in the survey and completed the first round (9 cardiologists (30%) and 23 anesthesiologists (57%)). Twenty five experts (78%) completed the second round (6 cardiologists and 19 anesthesiologists). One event suggested by an expert was added at the second round.

At the second round, Cronbach's a was 0.99 (95% CI 0.98–1.00) showing a high internal consistency indicating consensus between the experts and therefore the end of the Delphi process. Overall agreement between experts was good with an intra-class correlation coefficient at 0.72 (95% CI: 0.59–0.80).

### Consensus

A summary of experts' rating for each event and for each Delphi round is presented in Table 2. The ranking of the events slightly changed between the 1^st^ and 2^nd^ round. Events with the lowest rating of severity were: increased Troponin level (median = 3, Q1 = 3; Q3 = 4) and infra-popliteal venous thrombosis (median = 3, Q1 = 2; Q3 = 3). Events with the highest rating of importance were: ischemic stroke with severe disability at 7 days (median = 9, Q1 = 9; Q3 = 9), non-fatal myocardial infarction with heart failure (median = 9, Q1 = 8; Q3 = 9), massive pulmonary embolism (median = 9, Q1 = 9; Q3 = 9) and intra-cerebral hemorrhage with severe disability at 7 days (median = 9, Q1 = 9; Q3 = 9). Delphi-consensus weights are presented in Table 3. Ratings did not differ according to the specialty of experts (Appendix S1). Ratings at the first Delphi round did not differ between experts who responded at the second Delphi round and those who did not respond (Appendix S2).

**Table 2:** Summary of experts' rating at each Delphi round for the assessment of severity on a 10-point scale of events deriving from individual components of a composite outcome.

Events	Median (Q1–Q3) 1^st^ Delphi round	Median (Q1–Q3) 2^nd^ Delphi round
**Thombotic events:**		
Transcient ischemic attack	5 (3–6)	5 (4–5)
Ischemic stroke with no symptom at 7 days	6 (5–6)	6 (5–6)
Ischemic stroke with slight disability at 7 days	7 (6–7)	7 (6–7)
Ischemic stroke with moderate disability at 7 days	8 (7–8)	8 (8–8)
Ischemic stroke with severe disability at 7 days	9 (9–9)	9 (9–9)
Limb ischemia not requiring heparin or intervention	5 (4–6)	5 (4–5)
Limb ischemia requiring heparin or intervention	7 (6–7)	6 (6–7)
Limb ischemia requiring amputation	9 (8–9)	9 (8–9)
Increased level of troponin	4 (3–5)	4 (3–4)
Non-fatal myocardial infarction without heart failure	7 (6–8)	7 (6–7)
Non-fatal myocardial infarction with heart failure	8 (8-9)	9 (8–9)
Under-popliteal deep venous thrombosis	3 (2–4)	3 (2–3)
Deep venous thrombosis with iliac extension	6 (5–6)	6 (6–6)
Venous thrombosis of the pectoral limb	5 (4–6)	5 (4–5)
Venous thrombosis other	7 (6–8)	7 (6–7)
Pulmonary embolism	7 (6–8)	8 (7–8)
Massive pulmonary embolism	9 (8–9)	9 (9–9)
**Hemorrhagic events:**		
Intracerebral hemorrhage with no symptom at 7 days	6 (5–6)	6 (5–6)
Intracerebral hemorrhage with slight disability at 7 days	7 (7–7)	7 (7–7)
Intracerebral hemorrhage with moderate disability at 7 days	8 (8–8)	8 (8–8)
Intracerebral hemorrhage with severe disability at 7 days	9 (9–9)	9 (9–9)
Bleeding with increased length of stay		3 (2–4)
Bleeding requiring redo surgery or endoscopic sclerosis	5 (4–6)	5 (5–6)
Bleeding requiring both redosurgery and interventions to maintain cardiac output	7 (6–8)	7 (7–8)
Bleeding requiring transfusion of 3 U or more packed red blood cells	5 (4–7)	5 (4-6)
Bleeding requiring both transfusion of 3 U or more packed red blood cells and nterventions to increase cardiac output	7 (6–8)	7 (7–8)
Intra or retroperitoneal bleeding	6 (4–7)	6 (5–7)
Intra or retroperitoneal bleeding requiring interventions to maintain cardiac output	7 (6–8)	8 (7–8)

**Table 3:** Delphi-consensus weights for ischemic and bleeding events comprising a composite outcome in the field of thrombosis prevention.

Event	Rating
Death	10
Ischemic stroke with severe disability at 7 days	9
Limb ischemia requiring amputation	9
Non-fatal myocardial infarction with heart failure	9
Massive pulmonary embolism	9
Intracerebral hemorrhage with severe disability at 7 days	9
Ischemic stroke with moderate disability at 7 days	8
Pulmonary embolism	8
Intracerebral hemorrhage with moderate disability at 7 days	8
Intra or retroperitoneal bleeding requiring interventions to maintain cardiac output	8
Ischemic stroke with slight disability at 7 days	7
Non-fatal myocardial infarction without heart failure	7
Venous thrombosis other	7
Intracerebral hemorrhage with slight disability at 7 days	7
Bleeding requiring both redosurgery and interventions to maintain cardiac output	7
Bleeding requiring both transfusion of 3 U or more packed red blood cells and interventions to increase cardiac output	7
Ischemic stroke with no symptom at 7 days	6
Limb ischemia requiring heparin or intervention	6
Deep venous thrombosis with iliac extension	6
Intracerebral hemorrhage with no symptom at 7 days	6
Intra or retroperitoneal bleeding	6
Transcient ischemic attack	5
Limb ischemia not requiring heparin or intervention	5
Venous thrombosis of the pectoral limb	5
Bleeding requiring redo surgery or endoscopic sclerosis	5
Bleeding requiring transfusion of 3 U or more packed red blood cells	5
Increased level of troponin	4
Under-popliteal deep venous thrombosis	3
Bleeding with increased length of stay	3
No event	0

## Discussion

Before introducing a new treatment or strategy to common practice, or in comparative effectiveness research, capturing the overall impact of a therapeutic strategy in term of benefit and risk is important[25]. This is a well-recognized advantage of composite outcomes, but their use relies on the underlying assumption that patients will attach similar importance to each component [5]. However, this is rarely true. As outlined by Ferreira-Gonzalez[4] and cordoba[1], most composite end points showed either a large or moderate gradient in importance to patients. Weighting composite outcomes according to severity or importance to patients has been suggested to deal with this issue[8], [12]–[14]. This approach is possible only if a consensus can be reached on the importance of each individual component[15]. We report in this study how consensus-driven severity ratings were obtained for a wide range of ischemic and bleeding events comprising a composite outcome. The Delphi method was used to assign each individual component of the composite outcome a rating reflecting its severity. This well-recognized method to reach consensus in health care research[18]–[20] presents major advantages : it can be conducted via mail or email which improves feasibility and lowers costs and it can be completely anonymous which limits the influence of a single expert. Experts presented a high level of agreement so the Delphi was stopped at the second round.

All individual components of the composite outcome were ranked from the most (i.e., death) to the least severe (i.e., absence of event) considering the final median rating attributed by the experts for each event. There are several possibilities to deal with the fact that a single patient may present several events of interest during the follow-up period. As proposed by Braunwald[13], the score for each patient may represent the score of the most serious event encountered by this patient regardless of the number of events having occurred what we planned to do in this study. Another possibility could be to use the sum of the ratings for all outcomes encountered[14]. We believe that presenting both a transient ischemic attack (weight = 5) and increased level of troponin (weight = 4) during the follow-up period is not equivalent to ischemic stroke with severe disability at 7 days (weight = 9). Furthermore, we believe that death from myocardial infarction should not account for a higher rating than death from unknown cause occurring at home, which might also be due to myocardial infarction. Rating multiple events was not possible in our study given the number of possible combinations so the consensus was limited to severity ratings for each event and did not relate to their combination.

Felker proposed an alternative method[26]: all patients who met the worst event (i.e., death) during the follow-up would be assigned the worst ranks, in order to their time to event (e.g., the patient who died first would have the worst rank, the second patient who died the second worst rank). Patient not dying during study follow-up would be evaluated for the second worst endpoint and ranked above those who died, using the same methodology. Those patients not experiencing any of the event components during follow-up would be ranked according to quality of life scores from baseline to last follow-up. After all study subjects are ranked, the comparative efficacy of the 2 treatments is evaluated by comparing the ranks between the 2 groups.

Events rated by the experts to be included in the final composite outcome can be considered as patient important outcomes (which was previously defined as death, morbidity or, patient reported outcomes[27]). Nevertheless, a potential limitation of this study is the absence of involvement of patients to assess the severity of clinical events which may be differently perceived than by physicians. We believed that explaining clearly all events with their possible consequences to make the judgment of patients possible would have been difficult.

Whatever the way to use the ratings to build the composite outcome, there is no evidence that such a composite outcome represents a clinically meaningful endpoint. A validation study should be undertaken with comparison of the different strategies for integrating the ratings. Important questions may be also raised about which between-arm difference will be relevant, with implications for interpretation of results and sample size calculation. Calculating sample size is generally difficult for composite outcomes since information for the control group may be available for one or several components separately but rarely for the overall outcome. The most important problem pertains to the interpretation of results, which is not intuitive using this approach. Which between-arm difference for the final composite outcome corresponds to a clinically relevant difference is an issue.

It has to be noted that the severity ratings were ordinal and not true interval so the composite outcome should not theorically be treated as a continuous variable. We also made the assumption that the experts not responding at the second round would have had identical answers in the second round and applied a LOCF strategy. We compared the ratings at the first round between the experts having responded at the second round and those who did not and checked that there was no difference in the ratings (appendix S2). Third, we made the assumption that cardiologists and anesthesiologists would be consistent in their ratings, which we verified by comparing their ratings (appendix S1).

In conclusion, the consensus-driven ratings that were obtained could be used to weight ischemic and bleeding events of various severity comprising a composite outcome in the field of thrombosis prevention. This approach could be reproduced for other types of treatment and medical areas.

## Supporting Information

Appendix S1 Summary of experts' rating for the assessment of importance on a 10-point scale of events deriving from individual components of a composite outcome at the second Delphi round according to the specialty of experts.
(DOC)

Appendix S2 Comparison of summary of experts' rating at the first Delphi round between experts who responded at the second round and those who did not.
(DOC)
