# Supplementary material for: Delphi-Consensus Weights for Ischemic and Bleeding Events to Be Included in a Composite Outcome for RCTs in Thrombosis Prevention
Source: PLoS One. 2011 Apr 7;6(4):e18461. doi: 10.1371/journal.pone.0018461 (PMC3072399; doi:10.1371/journal.pone.0018461)
Supplement: Appendix S2 — Comparison of summary of experts' rating at the first Delphi round between experts who responded at the second round and those who did not. (DOC) [file pone.0018461.s002.doc]

Appendix S2: Comparison of summary of experts’ rating at the first Delphi round between experts who responded at the second round and those who did not

| **Events** | **Median (Q1-Q3)**  **Response** | **Median (Q1-Q3)**  **No response** |
| --- | --- | --- |
| **Thombotic events:**  Transcient ischemic attack  Ischemic stroke with no symptom at 7 days  Ischemic stroke with slight disability at 7 days  Ischemic stroke with moderate disability at 7 days  Ischemic stroke with severe disability at 7 days  Limb ischemia not requiring heparin or intervention  Limb ischemia requiring heparin or intervention  Limb ischemia requiring amputation  Increased level of troponin  Non-fatal myocardial infarction without heart failure  Non-fatal myocardial infarction with heart failure  Under-popliteal deep venous thrombosis  Deep venous thrombosis with iliac extension  Venous thrombosis of the pectoral limb  Venous thrombosis other  Pulmonary embolism  Massive pulmonary embolism | 5 (3-6)  6 (5-6)  7 (6-7)  8 (7-8)  9 (9-9)  5 (5-5)  7 (6-7)  9 (8-9)  3.5 (2-5)  7 (5-8)  9 (8-9)  3 (2-4)  5 (5-7)  5 (4-6)  6 (6-7)  7 (7-8)  9 (8-9) | 5 (4-5)  6 (5-6)  7 (6-8)  8 (7-8)  9 (9-9)  5 (4-6)  7 (5-7)  9 (8-9)  4 (3-5)  7 (6-7)  8 (8-9)  4 (4-5)  6 (6-6)  5 (4-6)  7 (5-8)  8 (7-8)  9 (9-10) |
| **Hemorrhagic events:**  Intracerebral hemorrhage with no symptom at 7 days  Intracerebral hemorrhage with slight disability at 7 days  Intracerebral hemorrhage with moderate disability at 7 days  Intracerebral hemorrhage with severe disability at 7 days  Bleeding with increased length of stay  Bleeding requiring redo surgery or endoscopic sclerosis  Bleeding requiring both redosurgery and interventions to maintain cardiac output  Bleeding requiring transfusion of 3 U or more packed red blood cells  Bleeding requiring both transfusion of 3 U or more packed red blood cells and  interventions to increase cardiac output  Intra or retroperitoneal bleeding  Intra or retroperitoneal bleeding requiring interventions to maintain cardiac output | 6 (5-6)  7 (6-7)  8 (7-8)  9 (9-9)  5 (4-6)  7 (6-8)  5 (4-7)  7 (5-8)  6 (3-6)  7 (5-8) | 6 (5-6)  7 (7-7)  8 (8-8)  9 (9-9)  5 (4-6)  7 (6-8)  7 (5-7)  8 (7-9)  7 (5-8)  8 (7-9) |
